# The 100 most influential papers on Lewy body dementias: a bibliometric analysis

**DOI:** 10.1097/MS9.0000000000002909

**Published:** 2025-01-31

**Authors:** Zain Ali Nadeem, Ahmad Danial, Zoya Imran, Adeena Jamil, Butool Nusrat, Sara Jawed, Amna Siddiqui, Unsa Habib Shaikh, Aalaa Saleh, Rameen Rao

**Affiliations:** a Department of Medicine, Allama Iqbal Medical College, Lahore, Pakistan; b Department of Medicine, Quaid-e-Azam Medical College, Bahawalpur, Pakistan; c Department of Medicine, Shalamar Medical and Dental College, Pakistan; d Department of Medicine, Dow International Medical College, Dow University of Health Sciences, Karachi, Pakistan; e Department of Medicine, Dow University of Health Sciences, Karachi, Pakistan; f Department of Medicine, Karachi Medical and Dental College, Pakistan; g Department of Medicine, Liaquat University of Health Sciences, Jamshoro, Sindh, Pakistan; h Faculty of Medicine, Lebanese University, Beirut, Lebanon; i Department of Medicine, Jinnah Sindh Medical University, Karachi, Pakistan

**Keywords:** bibliometric analysis, citation trends, dementia, Lewy body, Parkinson’s disease

## Abstract

**Introduction:**

Lewy body dementias (LBDs) are poorly understood neurodegenerative diseases. We aim to uncover the most cited articles and authors in the field, and to analyze the citations for gender differences.

**Methods:**

Two authors extracted the relevant articles from Scopus and ranked them according to the number of citations. Separate lists were prepared for the top 100 original articles and the top 15 review articles.

**Results:**

The 100 original studies were published from 1980 to 2019, with the greatest number published in the year 2000. The total citations ranged from 350 to 2640, with a median of 494.5. These articles originated from 17 countries, with major contributions from the USA (*n* = 31). While most of the first authors were men (*n* = 67), the citations per year were higher where the first authors when women. The last authors of the top 100 original articles were also predominantly male (*n* = 70). The greatest number of articles were published in neurology (*n* = 12) and brain (*n* = 11).

**Conclusion:**

Our results provide insights into the research trends and provide a list of the most influential papers on LBDs. Resolving the observed differences and promoting contribution from people all over the world is necessary to accelerate advancement in the field.

## Introduction

Lewy body dementias (LBD) include two conditions associated with aggregates of α-synuclein in the form of Lewy bodies in the brain: dementia with Lewy bodies and Parkinson’s disease dementia[1]. Given the lack of standardized assessment tools and the persisting controversies surrounding its diagnosis, it is a hotspot of research in dementias[2]. The precise etiology of LBD remains elusive, with theories revolving around neurotransmitters, inclusion bodies, and genetics providing fundamental insights. Associations have emerged between LBD and conditions like Parkinson’s disease, specific psychiatric illnesses, and various movement and behavior disorders. Despite this continuum, key differences persist, such as the unique distribution of α-synuclein in the brain in LBD compared to other disorders[3].

Recent studies have illuminated biomarkers, environmental factors, and the biology of dementia, enhancing diagnostic understanding and treatment strategies. However, the expanding body of research presents both valuable and conflicting evidence, possibly leading to overwhelming and inconclusive outcomes[4]. Evaluating existing scientific literature is a complex task. Bibliometric analysis utilizes quantitative methods on published scientific works to measure research activities in a specific field. Scientists leverage bibliometric analysis to reveal citation trends, assess article performance, and map collaboration patterns. It establishes a strong foundation for advancing a field by providing a comprehensive overview[5]. Citation analysis helps identify the most impactful papers in a field, facilitating researchers to delineate gaps in the existing literature and determine the direction for future research[6]. The primary purpose of this bibliometric analysis is to identify the cutting-edge papers, journals, and institutes concerning LBD research, the citation trends, and the funding landscape. Additionally, no previous studies could be identified that assessed the gender distribution of authors in this field. As such, our secondary objective was to demarcate any associations, or the lack thereof, of the gender of the lead and senior authors on the papers and their citation trends. This would help gauge the state of gender equity in the field.

## Materials and methods

This manuscript adheres to the Preliminary guideline for reporting bibliometric reviews of the biomedical literature (BIBLIO)[7].

### Data sources

We searched Scopus for all articles regarding the LBDs from inception till 20 June 2023. As the purpose of our paper was to compare citations, we needed to use a citation index – a database that keeps track of the number of citations of each article. Multiple databases cannot be searched due to differences in indexing and citations counts, whereby the citations might be slightly different in one database than others. Traditionally, Web of Science has been used for this purpose. However, we selected Scopus due to its more reliable citation counts and broader coverage of journals: Scopus gives around 20% greater coverage than Web of Science[8]. PubMed (MEDLINE) and Embase do not index citation counts, and Google Scholar has been concluded to be unreliable in the context of bibliometric analysis[9]. Ethical approval was not required for the study, as only previously published data was analyzed.

### Article search and selection

The search was conducted using the following string: (TITLE-ABS-KEY (dementia AND “lewy body”) OR TITLE-ABS-KEY (“parkinson’s disease dementia”) OR TITLE-ABS-KEY (“lewy body disease”)). We included articles if they: (1) were original studies or review articles; (2) pertained to LBDs, either in the context of dementia with Lewy bodies or Parkinson’s disease dementia; (3) were human, animal, or laboratory studies. Human studies needed to have recruited patients with a diagnosis of DLB – either dementia with Lewy bodies or Parkinson’s disease dementia – while animal and laboratory studies needed to have studied the mechanisms behind either of these conditions. We also included papers on REM sleep behavior disorder (RBD) only if they specifically focused on the risk of progressing from RBD to LBD or included patients with RBD who were also diagnosed with LBD. As this criterion could not be applied to review articles, reviews were included if they had any discussion of Parkinson’s disease dementia or dementia with Lewy Bodies in the full text; this meant the reviews might not be as specific as the original articles, so the review articles were not included in the statistical analysis. We excluded articles if they were: (1) letters, editorials, or case reports; (2) did not pertain to Lewy bodies, but to other synucleinopathies; or (3) included Parkinson’s disease patients without dementia. The original articles were imported to Mendeley reference manager and manually screened by two authors independently using the titles and abstracts. The results of the title and abstract screening were matched by the two reviewers, and disagreements were resolved by mutual discussion until a consensus was reached. Afterwards, the full texts of potentially relevant articles were reviewed and matched against the eligibility criteria, and the articles were again matched by the two reviewers, with disagreements resolved by mutual consensus. The relevant original articles were then arranged in descending order of citations to create a list of the top 100 most-cited original articles. The same screening strategy was used for review articles and a list of the top 15 most-cited review articles was prepared.

### Data abstraction

The following were extracted or calculated for the top 100 original articles: (1) rank, (2) title, (3) year of publication, (4) total citations, (5) average citations per year, (6) names of authors with their H-indices, (7) first author’s affiliation at the time of publication, (8) first and last author’s gender, (9) name of journal with impact factor, (10) country of origin, (11) conflict of interest, (12) funding, and (13) type of subjects. The first author’s country and institute were noted for articles with authors from different countries or institutes. The genders of the first and last authors were identified from the pronouns used in professional profiles; he/him was considered “male”, she/her was considered “female”, and any other pronouns were noted as “other”. The authors were contacted when gender or pronouns were not mentioned in the profile, and the gender was noted as “not retrieved” if no response was received from the authors. The journal impact factors were obtained from Journal Citation Reports (Clarivate, 2022). The subjects were classified into “human”, “animals”, and “ex-vivo”; ex-vivo included all laboratory studies.

### Data analysis

We checked the quantitative data for normality using the Kolmogorov-Smirnov test. Subsequently, we described normal data using means and standard deviations (SDs) and skewed data using medians and interquartile ranges (IQRs). The analysis was conducted on IBM SPSS version 26. A two-sided *P*-value < 0.05 was considered significant in all cases.

## Results

### Total citations, citations per year, and type of subjects

**Figure 1.**
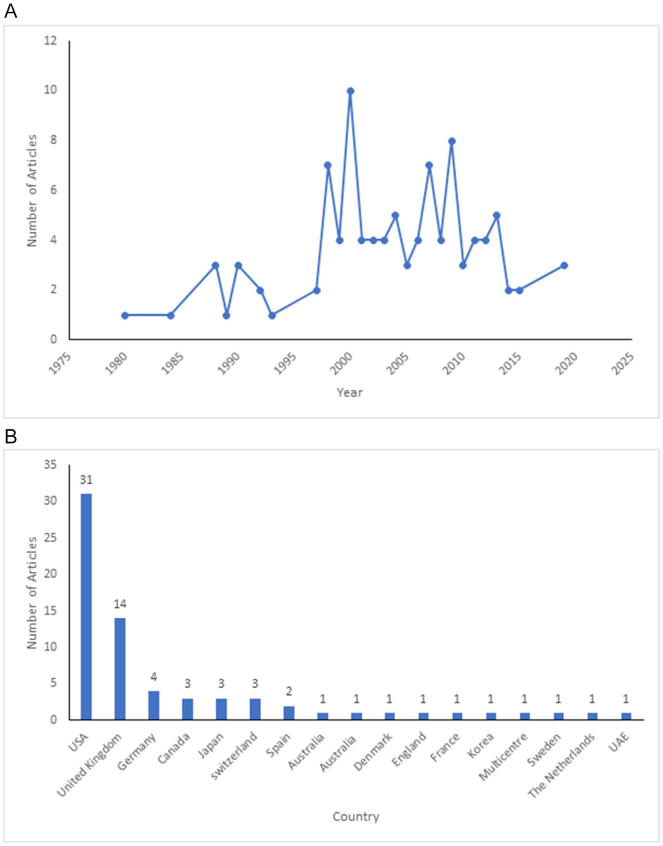
Distribution of original articles by (A) year of publication and (B) country of origin.

The 100 most cited original articles that matched our eligibility criteria are mentioned in Table 1. The most cited article was “α-Synuclein in filamentous inclusions of Lewy bodies from Parkinson’s disease and dementia with Lewy bodies” published in Proceedings of the National Academy of Sciences of the United States of America in 1998. The greatest citations per year belonged to the article “Transneuronal Propagation of Pathologic α-Synuclein from the Gut to the Brain Models Parkinson’s Disease” published in Neuron in 2019. Figure 1A shows the trend of total citations of the 100 articles according to year. The highest number of citations was seen between 1990 and 2000. Humans were the most studied subjects (*n* = 72), followed by equal number of animal studies (*n* = 14) and ex-vivo studies (*n* = 14). The total number of citations for the original articles was 63 741, with a median of 494.5 (IQR 275) citations per article. Each article was cited a median of 28.2 citations per year (IQR 33).

**Table 1:** The Top 100 most-cited original articles on dementias with Lewy bodies

Rank	AMA citation	Year	Total citations	Citations per year	Type of subjects
1	Spillantini MG, Crowther RA, Jakes R, Hasegawa M, Goedert M. alpha-Synuclein in filamentous inclusions of Lewy bodies from Parkinson’s disease and dementia with Lewy bodies. Proc Natl Acad Sci U S A. 1998;95(11):6469-6473. doi:10.1073/pnas.95.11.6469	1998	2460	98.4	Humans
2	Zimprich A, Biskup S, Leitner P, et al. Mutations in LRRK2 cause autosomal-dominant parkinsonism with pleomorphic pathology. Neuron. 2004;44(4):601-607. doi:10.1016/j.neuron.2004.11.005	2004	2332	122.74	Humans
3	McGeer PL, Itagaki S, Boyes BE, McGeer EG. Reactive microglia are positive for HLA-DR in the substantia nigra of Parkinson’s and Alzheimer’s disease brains. Neurology. 1988;38(8):1285-1291. doi:10.1212/wnl.38.8.1285	1988	2295	65.57	Humans
4	Masliah E, Rockenstein E, Veinbergs I, et al. Dopaminergic loss and inclusion body formation in alpha-synuclein mice: implications for neurodegenerative disorders. Science. 2000;287(5456):1265-1269. doi:10.1126/science.287.5456.1265	2000	1577	68.57	Animals
5	Fujiwara H, Hasegawa M, Dohmae N, et al. alpha-Synuclein is phosphorylated in synucleinopathy lesions. Nat Cell Biol. 2002;4(2):160-164. doi:10.1038/ncb748	2002	1537	73.19	Humans
6	Mioshi E, Dawson K, Mitchell J, Arnold R, Hodges JR. The Addenbrooke’s Cognitive Examination Revised (ACE-R): a brief cognitive test battery for dementia screening. Int J Geriatr Psychiatry. 2006;21(11):1078-1085. doi:10.1002/gps.1610	2006	1436	84.47	Humans
7	McKee AC, Stern RA, Nowinski CJ, et al. The spectrum of disease in chronic traumatic encephalopathy [published correction appears in Brain. 2013 Oct;136(Pt 10):e255]. Brain. 2013;136(Pt 1):43-64. doi:10.1093/brain/aws307	2013	1434	143.4	Humans
8	Baba M, Nakajo S, Tu PH, et al. Aggregation of alpha-synuclein in Lewy bodies of sporadic Parkinson’s disease and dementia with Lewy bodies. Am J Pathol. 1998;152(4):879-884.	1998	1417	56.68	Humans
9	Giasson BI, Duda JE, Murray IV, et al. Oxidative damage linked to neurodegeneration by selective alpha-synuclein nitration in synucleinopathy lesions. Science. 2000;290(5493):985-989. doi:10.1126/science.290.5493.985	2000	1405	61.09	Humans
10	Schneider JA, Arvanitakis Z, Bang W, Bennett DA. Mixed brain pathologies account for most dementia cases in community-dwelling older persons. Neurology. 2007;69(24):2197-2204. doi:10.1212/01.wnl.0000271090.28148.24	2007	1290	80.63	Humans
11	McKeith I, Del Ser T, Spano P, et al. Efficacy of rivastigmine in dementia with Lewy bodies: a randomised, double-blind, placebo-controlled international study. Lancet. 2000;356(9247):2031-2036. doi:10.1016/S0140-6736(00)03399-7	2000	1076	46.78	Humans
12	Volpicelli-Daley LA, Luk KC, Patel TP, et al. Exogenous α-synuclein fibrils induce Lewy body pathology leading to synaptic dysfunction and neuron death. Neuron. 2011;72(1):57-71. doi:10.1016/j.neuron.2011.08.033	2011	1015	84.58	Humans
13	Uversky VN, Li J, Fink AL. Evidence for a partially folded intermediate in alpha-synuclein fibril formation. J Biol Chem. 2001;276(14):10737-10744. doi:10.1074/jbc.M010907200	2001	952	43.27	Ex-vivo
14	Anderson JP, Walker DE, Goldstein JM, et al. Phosphorylation of Ser-129 is the dominant pathological modification of alpha-synuclein in familial and sporadic Lewy body disease. J Biol Chem. 2006;281(40):29739-29752. doi:10.1074/jbc.M600933200	2006	943	55.47	Ex-vivo
15	Rowe CC, Ng S, Ackermann U, et al. Imaging beta-amyloid burden in aging and dementia. Neurology. 2007;68(20):1718-1725. doi:10.1212/01.wnl.0000261919.22630.ea	2007	931	58.19	Humans
16	Hughes AJ, Daniel SE, Blankson S, Lees AJ. A clinicopathologic study of 100 cases of Parkinson’s disease. Arch Neurol. 1993;50(2):140-148. doi:10.1001/archneur.1993.00540020018011	1993	846	28.2	Humans
17	Peelaerts W, Bousset L, Van der Perren A, et al. α-Synuclein strains cause distinct synucleinopathies after local and systemic administration. Nature. 2015;522(7556):340-344. doi:10.1038/nature14547	2015	797	99.63	Animals
18	Olson EJ, Boeve BF, Silber MH. Rapid eye movement sleep behaviour disorder: demographic, clinical and laboratory findings in 93 casesSilber MH. Brain. 2000;123 (Pt 2):331-339. doi:10.1093/brain/123.2.331	2000	789	34.3	Humans
19	Luk KC, Kehm VM, Zhang B, O’Brien P, Trojanowski JQ, Lee VM. Intracerebral inoculation of pathological α-synuclein initiates a rapidly progressive neurodegenerative α-synucleinopathy in mice. J Exp Med. 2012;209(5):975-986. doi:10.1084/jem.20112457	2012	772	70.18	Animals
20	Iranzo A, Molinuevo JL, Santamaría J, et al. Rapid-eye-movement sleep behaviour disorder as an early marker for a neurodegenerative disorder: a descriptive study. Lancet Neurol. 2006;5(7):572-577. doi:10.1016/S1474-4422(06)70476-8	2006	769	45.24	Humans
21	Williams-Gray CH, Foltynie T, Brayne CE, Robbins TW, Barker RA. Evolution of cognitive dysfunction in an incident Parkinson’s disease cohort. Brain. 2007;130(Pt 7):1787-1798. doi:10.1093/brain/awm111	2007	729	45.56	Humans
22	Williams-Gray CH, Evans JR, Goris A, et al. The distinct cognitive syndromes of Parkinson’s disease: 5 year follow-up of the CamPaIGN cohort. Brain. 2009;132(Pt 11):2958-2969. doi:10.1093/brain/awp245	2009	700	50	Humans
23	Beach TG, Adler CH, Sue LI, et al. Multi-organ distribution of phosphorylated alpha-synuclein histopathology in subjects with Lewy body disorders. Acta Neuropathol. 2010;119(6):689-702. doi:10.1007/s00401-010-0664-3	2010	685	52.69	Humans
24	Chung KK, Thomas B, Li X, et al. S-nitrosylation of parkin regulates ubiquitination and compromises parkin’s protective function. Science. 2004;304(5675):1328-1331. doi:10.1126/science.1093891	2004	677	35.63	Animals
25	Ferrante RJ, Browne SE, Shinobu LA, et al. Evidence of increased oxidative damage in both sporadic and familial amyotrophic lateral sclerosis. J Neurochem. 1997;69(5):2064-2074. doi:10.1046/j.1471-4159.1997.69052064.x	1997	667	25.65	Humans
26	Serpell LC, Berriman J, Jakes R, Goedert M, Crowther RA. Fiber diffraction of synthetic alpha-synuclein filaments shows amyloid-like cross-beta conformation. Proc Natl Acad Sci U S A. 2000;97(9):4897-4902. doi:10.1073/pnas.97.9.4897	2000	664	28.87	Ex-vivo
27	Kim S, Kwon SH, Kam TI, et al. Transneuronal propagation of pathologic α-synuclein from the gut to the brain models parkinson’s disease. Neuron. 2019;103(4):627-641.e7. doi:10.1016/j.neuron.2019.05.035	2019	650	162.5	Animals
28	Postuma RB, Gagnon JF, Vendette M, Fantini ML, Massicotte-Marquez J, Montplaisir J. Quantifying the risk of neurodegenerative disease in idiopathic REM sleep behavior disorder. Neurology. 2009;72(15):1296-1300. doi:10.1212/01.wnl.0000340980.19702.6e	2009	633	45.21	Humans
29	Lee HJ, Suk JE, Patrick C, et al. Direct transfer of alpha-synuclein from neuron to astroglia causes inflammatory responses in synucleinopathies. J Biol Chem. 2010;285(12):9262-9272. doi:10.1074/jbc.M109.081125	2010	614	47.23	Animals
30	Danzer KM, Kranich LR, Ruf WP, et al. Exosomal cell-to-cell transmission of alpha synuclein oligomers. Mol Neurodegener. 2012;7:42. Published 24 August 2012. doi:10.1186/1750-1326-7-42	2012	610	55.45	Ex-vivo
31	Bousset L, Pieri L, Ruiz-Arlandis G, et al. Structural and functional characterization of two alpha-synuclein strains. Nat Commun. 2013;4:2575. doi:10.1038/ncomms3575	2013	593	59.3	Ex-vivo
32	El-Agnaf OM, Salem SA, Paleologou KE, et al. Detection of oligomeric forms of alpha-synuclein protein in human plasma as a potential biomarker for Parkinson’s disease. FASEB J. 2006;20(3):419-425. doi:10.1096/fj.03-1449com	2006	591	34.76	Humans
33	Masuda-Suzukake M, Nonaka T, Hosokawa M, et al. Prion-like spreading of pathological α-synuclein in brain. Brain. 2013;136(Pt 4):1128-1138. doi:10.1093/brain/awt037IF: 14.5 Q1	2013	589	58.9	Animals
34	Schenck CH, Boeve BF, Mahowald MW. Delayed emergence of a Parkinsonian disorder or dementia in 81% of older men initially diagnosed with idiopathic rapid eye movement sleep behavior disorder: a 16-year update on a previously reported series. Sleep Med. 2013;14(8):744-748. doi:10.1016/j.sleep.2012.10.009	2013	585	58.5	Humans
35	Perry RH, Irving D, Blessed G, Fairbairn A, Perry EK. Senile dementia of Lewy body type. A clinically and neuropathologically distinct form of Lewy body dementia in the elderly. J Neurol Sci. 1990;95(2):119-139. doi:10.1016/0022-510x(90)90236-g	1990	564	17.09	Humans
36	McKeith I, Fairbairn A, Perry R, Thompson P, Perry E. Neuroleptic sensitivity in patients with senile dementia of Lewy body type. BMJ. 1992;305(6855):673-678. doi:10.1136/bmj.305.6855.673	1992	555	17.9	Humans
37	Mosconi L, Tsui WH, Herholz K, et al. Multicenter standardized 18 F-FDG PET diagnosis of mild cognitive impairment, Alzheimer’s disease, and other dementias. J Nucl Med. 2008;49(3):390-398. doi:10.2967/jnumed.107.045385	2008	548	36.53	Humans
38	Zhang J, Perry G, Smith MA, et al. Parkinson’s disease is associated with oxidative damage to cytoplasmic DNA and RNA in substantia nigra neurons. Am J Pathol. 1999;154(5):1423-1429. doi:10.1016/S0002-9440(10)65396-5	1999	537	22.38	Humans
39	Neumann J, Bras J, Deas E, et al. Glucocerebrosidase mutations in clinical and pathologically proven Parkinson’s disease. Brain. 2009;132(Pt 7):1783-1794. doi:10.1093/brain/awp044	2009	535	38.21	Humans
40	Hurtig HI, Trojanowski JQ, Galvin J, et al. Alpha-synuclein cortical Lewy bodies correlate with dementia in Parkinson’s disease. Neurology. 2000;54(10):1916-1921. doi:10.1212/wnl.54.10.1916	2000	522	22.7	Humans
41	Barker WW, Luis CA, Kashuba A, et al. Relative frequencies of Alzheimer disease, Lewy body, vascular and frontotemporal dementia, and hippocampal sclerosis in the State of Florida Brain Bank. Alzheimer Dis Assoc Disord. 2002;16(4):203-212. doi:10.1097/00002093-200210000-00001	2002	522	24.86	Humans
42	Andreasen N, Minthon L, Davidsson P, et al. Evaluation of CSF-tau and CSF-Abeta42 as diagnostic markers for Alzheimer disease in clinical practice. Arch Neurol. 2001;58(3):373-379. doi:10.1001/archneur.58.3.373	2001	517	23.5	Humans
43	Iranzo A, Tolosa E, Gelpi E, et al. Neurodegenerative disease status and post-mortem pathology in idiopathic rapid-eye-movement sleep behaviour disorder: an observational cohort study. Lancet Neurol. 2013;12(5):443-453. doi:10.1016/S1474-4422(13)70056-5	2013	515	51.5	Humans
44	Jo E, McLaurin J, Yip CM, St George-Hyslop P, Fraser PE. alpha-Synuclein membrane interactions and lipid specificity. J Biol Chem. 2000;275(44):34328-34334. doi:10.1074/jbc.M004345200	2000	514	22.35	Humans
45	Burton EJ, McKeith IG, Burn DJ, Williams ED, O’Brien JT. Cerebral atrophy in Parkinson’s disease with and without dementia: a comparison with Alzheimer’s disease, dementia with Lewy bodies and controls. Brain. 2004;127(Pt 4):791-800. doi:10.1093/brain/awh088	2004	512	26.95	Humans
46	Spencer B, Potkar R, Trejo M, et al. Beclin 1 gene transfer activates autophagy and ameliorates the neurodegenerative pathology in alpha-synuclein models of Parkinson’s and Lewy body diseases. J Neurosci. 2009;29(43):13578-13588. doi:10.1523/JNEUROSCI.4390-09.2009	2009	505	36.07	Animals
47	Mollenhauer B, Locascio JJ, Schulz-Schaeffer W, Sixel-Döring F, Trenkwalder C, Schlossmacher MG. α-Synuclein and tau concentrations in cerebrospinal fluid of patients presenting with parkinsonism: a cohort study [published correction appears in Lancet Neurol. 2011 Apr;10(4):297]. Lancet Neurol. 2011;10(3):230-240. doi:10.1016/S1474-4422(11)70014-X	2011	504	42	Humans
48	Lu T, Aron L, Zullo J, et al. REST and stress resistance in ageing and Alzheimer’s disease [published correction appears in Nature. 15 December 2016;540(7633):470]. Nature. 2014;507(7493):448-454. doi:10.1038/nature13163	2014	502	55.78	Humans
49	McKeith I, O’Brien J, Walker Z, et al. Sensitivity and specificity of dopamine transporter imaging with 123I-FP-CIT SPECT in dementia with Lewy bodies: a phase III, multicentre study. Lancet Neurol. 2007;6(4):305-313. doi:10.1016/S1474-4422(07)70057-1	2007	501	31.31	Humans
50	Harding, A. J., Broe, G. A., & Halliday, G. M. (2002). Visual hallucinations in Lewy body disease relate to Lewy bodies in the temporal lobe. Brain, 125(2), 391–403. doi:10.1093/brain/awf033	2002	501	23.86	Humans
51	Alam, Z. I., Daniel, S. E., Lees, A. J., Marsden, D. C., Jenner, P., & Halliwell, B. (2002). A generalised increase in protein carbonyls in the brain in Parkinson’s but not incidental Lewy body disease. Journal of Neurochemistry, 69(3), 1326–1329. doi:10.1046/j.1471-4159.1997.69031326.x	1997	488	18.77	Humans
52	Van der Putten, H., Wiederhold, K.-H., Probst, A., Barbieri, S., Mistl, C., Danner, S., … Bilbe, G. (2000). Neuropathology in mice expressing human α-synuclein. The Journal of Neuroscience, 20(16), 6021–6029. doi:10.1523/jneurosci.20-16-06021.2000	2000	482	20.96	Animals
53	Beach, T. G., Adler, C. H., Lue, L., Sue, L. I., Bachalakuri, J., … Walker, D. G. (2009). Unified staging system for Lewy body disorders: correlation with nigrostriatal degeneration, cognitive impairment and motor dysfunction. Acta Neuropathologica, 117(6), 613–634. doi:10.1007/s00401-009-0538-8	2009	478	34.14	Humans
54	Postuma, R. B., Iranzo, A., Hu, M., Högl, B., Boeve, B. F., Manni, R., … Puligheddu, M. (2019). Risk and predictors of dementia and parkinsonism in idiopathic REM sleep behaviour disorder: a multicentre study. Brain. doi:10.1093/brain/awz030	2019	478	119.5	Humans
55	Sharon, R., Bar-Joseph, I., Frosch, M. P., Walsh, D. M., Hamilton, J. A., & Selkoe, D. J. (2003). The formation of highly soluble oligomers of α-synuclein is regulated by fatty acids and enhanced in Parkinson’s disease. Neuron, 37(4), 583–595. doi:10.1016/s0896-6273(03)00024-2	2003	478	23.9	Humans
56	Masliah, E., Rockenstein, E., Adame, A., Alford, M., Crews, L., Hashimoto, M., … Schenk, D. (2005). Effects of α-synuclein immunization in a mouse model of Parkinson’s disease. Neuron, 46(6), 857–868. doi:10.1016/j.neuron.2005.05.0	2005	477	26.5	Animals
57	El-Agnaf, O. M. A., Salem, S. A., Paleologou, K. E., COOPER, L. J., Fullwood, N. J., Gibson, M. J., … Allsop, D. (2003). α-Synuclein implicated in Parkinson’s disease is present in extracellular biological fluids, including human plasma. The FASEB Journal, 17(13), 1945–1947. doi:10.1096/fj.03-0098fje	2003	477	23.85	Ex-vivo
58	Lowe, J., Blanchard, A., Morrell, K., Lennox, G., Reynolds, L., Billett, M., … Mayer, R. J. (1988). Ubiquitin is a common factor in intermediate filament inclusion bodies of diverse type in man, including those of Parkinson’s disease, Pick’s disease, and Alzheimer’s disease, as well as Rosenthal fibres in cerebellar astrocytomas, cytoplasmic bodies in muscle, and mallory bodies in alcoholic liver disease. The Journal of Pathology, 155(1), 9–15. doi:10.1002/path.1711550105	1988	472	13.49	Ex-vivo
59	Jensen, P. H., Nielsen, M. S., Jakes, R., Dotti, C. G., & Goedert, M. (1998). Binding of α-synuclein to brain vesicles is abolished by familial Parkinson’s disease mutation. Journal of Biological Chemistry, 273(41), 26292–26294. doi:10.1074/jbc.273.41.26292	1998	466	18.64	Animals
60	Gitler, A. D., Chesi, A., Geddie, M. L., Strathearn, K. E., Hamamichi, S., Hill, K. J., … Lindquist, S. (2009). α-Synuclein is part of a diverse and highly conserved interaction network that includes PARK9 and manganese toxicity. Nature Genetics, 41(3), 308–315. doi:10.1038/ng.300	2009	461	32.93	Ex-vivo
61	Marquié, M., Normandin, M. D., Vanderburg, C. R., Costantino, I. M., Bien, E. A., Rycyna, L. G., … Gómez-Isla, T. (2015). Validating novel tau positron emission tomography tracer [F-18]-AV-1451 (T807) on postmortem brain tissue. Annals of Neurology, 78(5), 787–800. doi:10.1002/ana.24517	2015	461	57.63	Ex-vivo
62	Davis DG, Schmitt FA, Wekstein DR, Markesbery WR. Alzheimer neuropathologic alterations in aged cognitively normal subjects. J Neuropathol Exp Neurol. 1999;58(4):376-388. doi:10.1097/00005072-199904000-00008	1999	458	19.08	Humans
63	Minoshima S, Foster NL, Sima AA, Frey KA, Albin RL, Kuhl DE. Alzheimer’s disease versus dementia with Lewy bodies: cerebral metabolic distinction with autopsy confirmation. Ann Neurol. 2001;50(3):358-365. doi:10.1002/ana.1133	2001	454	20.64	Humans
64	Boller F, Mizutani T, Roessmann U, Gambetti P. Parkinson disease, dementia, and Alzheimer disease: clinicopathological correlations. Ann Neurol. 1980;7(4):329-335. doi:10.1002/ana.410070408	1980	454	10.56	Ex-vivo
65	Bennett DA, Schneider JA, Bienias JL, Evans DA, Wilson RS. Mild cognitive impairment is related to Alzheimer disease pathology and cerebral infarctions. Neurology. 2005;64(5):834-841. doi:10.1212/01.WNL.0000152982.47274.9E	2005	452	25.11	Humans
66	Shahmoradian SH, Lewis AJ, Genoud C, et al. Lewy pathology in Parkinson’s disease consists of crowded organelles and lipid membranes. Nat Neurosci. 2019;22(7):1099-1109. doi:10.1038/s41593-019-0423-2	2019	438	109.5	Humans
67	Braak H, Rüb U, Jansen Steur EN, Del Tredici K, de Vos RA. Cognitive status correlates with neuropathologic stage in Parkinson disease. Neurology. 2005;64(8):1404-1410. doi:10.1212/01.WNL.0000158422.41380.82	2005	437	24.28	Humans
68	Kramer ML, Schulz-Schaeffer WJ. Presynaptic alpha-synuclein aggregates, not Lewy bodies, cause neurodegeneration in dementia with Lewy bodies. J Neurosci. 2007;27(6):1405-1410. doi:10.1523/JNEUROSCI.4564-06.2007	2007	432	27	Ex-vivo
69	Klucken J, Shin Y, Masliah E, Hyman BT, McLean PJ. Hsp70 reduces alpha-synuclein aggregation and toxicity. J Biol Chem. 2004;279(24):25497-25502. doi:10.1074/jbc.M400255200	2004	428	22.53	Animals
70	Chan-Palay V, Asan E. Alterations in catecholamine neurons of the locus coeruleus in senile dementia of the Alzheimer type and in Parkinson’s disease with and without dementia and depression. J Comp Neurol. 1989;287(3):373-392. doi:10.1002/cne.902870308	1989	424	12.47	Humans
71	Kosaka K, Yoshimura M, Ikeda K, Budka H. Diffuse type of Lewy body disease: progressive dementia with abundant cortical Lewy bodies and senile changes of varying degree – a new disease? Clin Neuropathol. 1984;3(5):185-192.	1984	423	10.85	Humans
72	McKeith IG, Perry RH, Fairbairn AF, Jabeen S, Perry EK. Operational criteria for senile dementia of Lewy body type (SDLT). Psychol Med. 1992;22(4):911-922. doi:10.1017/s0033291700038484	1992	418	13.48	Humans
73	Compta Y, Parkkinen L, O’Sullivan SS, et al. McKeith IG, Perry RH, Fairbairn AF, Jabeen S, Perry EK. Lewy- and Alzheimer-type pathologies in Parkinson’s disease dementia: which is more important? Brain. 2011;134(Pt 5):1493-1505. doi:10.1093/brain/awr031	2011	412	34.33	Humans
74	Aarsland D, Ballard C, Walker Z, et al. Memantine in patients with Parkinson’s disease dementia or dementia with Lewy bodies: a double-blind, placebo-controlled, multicentre trial. Lancet Neurol. 2009;8(7):613-618. doi:10.1016/S1474-4422(09)70146-2	2009	393	28.07	Humans
75	McKeith IG, Ballard CG, Perry RH, et al. Prospective validation of consensus criteria for the diagnosis of dementia with Lewy bodies. Neurology. 2000;54(5):1050-1058. doi:10.1212/wnl.54.5.1050	2000	392	17.04	Humans
76	Clinton LK, Blurton-Jones M, Myczek K, Trojanowski JQ, LaFerla FM. Synergistic Interactions between Abeta, tau, and alpha-synuclein: acceleration of neuropathology and cognitive decline. J Neurosci. 2010;30(21):7281-7289. doi:10.1523/JNEUROSCI.0490-10.2010	2010	391	30.08	Animals
77	Gomperts SN, Rentz DM, Moran E, et al. Imaging amyloid deposition in Lewy body diseases. Neurology. 2008;71(12):903-910. doi:10.1212/01.wnl.0000326146.60732.d6	2008	391	26.07	Humans
78	Rockenstein E, Mallory M, Hashimoto M, et al. Differential neuropathological alterations in transgenic mice expressing alpha-synuclein from the platelet-derived growth factor and Thy-1 promoters. J Neurosci Res. 2002;68(5):568-578. doi:10.1002/jnr.10231	2002	386	18.38	Animals
79	Orimo S, Uchihara T, Nakamura A, et al. Axonal alpha-synuclein aggregates herald centripetal degeneration of cardiac sympathetic nerve in Parkinson’s disease. Brain. 2008;131(Pt 3):642-650. doi:10.1093/brain/awm302	2008	378	25.2	Humans
80	Lobotesis K, Fenwick JD, Phipps A, et al. Occipital hypoperfusion on SPECT in dementia with Lewy bodies but not AD. Neurology. 2001;56(5):643-649. doi:10.1212/wnl.56.5.643	2001	376	17.09	Humans
81	Galvin JE, Uryu K, Lee VM, Trojanowski JQ. Axon pathology in Parkinson’s disease and Lewy body dementia hippocampus contains alpha-, beta-, and gamma-synuclein. Proc Natl Acad Sci U S A. 1999;96(23):13450-13455. doi:10.1073/pnas.96.23.13450	1999	374	15.58	Humans
82	Boeve BF, Silber MH, Ferman TJ, et al. REM sleep behavior disorder and degenerative dementia: an association likely reflecting Lewy body disease. Neurology. 1998;51(2):363-370. doi:10.1212/wnl.51.2.363	1998	374	14.96	Humans
83	Takeda A, Mallory M, Sundsmo M, Honer W, Hansen L, Masliah E. Abnormal accumulation of NACP/alpha-synuclein in neurodegenerative disorders. Am J Pathol. 1998;152(2):367-372.	1998	371	14.84	Humans
84	Edison P, Rowe CC, Rinne JO, et al. Amyloid load in Parkinson’s disease dementia and Lewy body dementia measured with [11C]PIB positron emission tomography. J Neurol Neurosurg Psychiatry. 2008;79(12):1331-1338. doi:10.1136/jnnp.2007.127878	2008	365	24.33	Humans
85	Irizarry MC, Growdon W, Gomez-Isla T, et al. Nigral and cortical Lewy bodies and dystrophic nigral neurites in Parkinson’s disease and cortical Lewy body disease contain alpha-synuclein immunoreactivity. J Neuropathol Exp Neurol. 1998;57(4):334-337. doi:10.1097/00005072-199804000-00005	1998	364	14.56	Humans
86	Hampel H, Buerger K, Zinkowski R, et al. Measurement of phosphorylated tau epitopes in the differential diagnosis of Alzheimer disease: a comparative cerebrospinal fluid study. Arch Gen Psychiatry. 2004;61(1):95-102. doi:10.1001/archpsyc.61.1.95	2004	363	19.11	Humans
87	Devine MJ, Ryten M, Vodicka P, et al. Parkinson’s disease induced pluripotent stem cells with triplication of the α-synuclein locus. Nat Commun. 2011;2:440. Published 23 August 2011. doi:10.1038/ncomms1453	2011	363	30.25	Ex-vivo
88	Golbe LI, Di Iorio G, Bonavita V, Miller DC, Duvoisin RC. A large kindred with autosomal dominant Parkinson’s disease. Ann Neurol. 1990;27(3):276-282. doi:10.1002/ana.410270309	1990	358	10.85	Humans
89	Hall S, Öhrfelt A, Constantinescu R, et al. Accuracy of a panel of 5 cerebrospinal fluid biomarkers in the differential diagnosis of patients with dementia and/or parkinsonian disorders. Arch Neurol. 2012;69(11):1445-1452. doi:10.1001/archneurol.2012.1654	2012	357	32.45	Humans
90	Kuzuhara S, Mori H, Izumiyama N, Yoshimura M, Ihara Y. Lewy bodies are ubiquitinated. A light and electron microscopic immunocytochemical study. Acta Neuropathol. 1988;75(4):345-353. doi:10.1007/BF00687787	1988	357	10.2	Ex-vivo
91	Barber R, Scheltens P, Gholkar A, et al. White matter lesions on magnetic resonance imaging in dementia with Lewy bodies, Alzheimer’s disease, vascular dementia, and normal aging. J Neurol Neurosurg Psychiatry. 1999;67(1):66-72. doi:10.1136/jnnp.67.1.66	1999	357	14.88	Humans
92	Crowther RA, Jakes R, Spillantini MG, Goedert M. Synthetic filaments assembled from C-terminally truncated alpha-synuclein. FEBS Lett. 1998;436(3):309-312. doi:10.1016/s0014-5793(98)01146-6	1998	356	14.24	Ex-vivo
93	Boeve BF, Silber MH, Parisi JE, et al. Synucleinopathy pathology and REM sleep behavior disorder plus dementia or parkinsonism. Neurology. 2003;61(1):40-45. doi:10.1212/01.wnl.0000073619.94467.b0	2003	355	17.75	Humans
94	Iranzo A, Fernández-Arcos A, Tolosa E, et al. Neurodegenerative disorder risk in idiopathic REM sleep behavior disorder: study in 174 patients. PLoS One. 2014;9(2):e89741. Published 26 February 2014. doi:10.1371/journal.pone.0089741	2014	354	39.33	Humans
95	Boeve BF, Silber MH, Ferman TJ. Melatonin for treatment of REM sleep behavior disorder in neurologic disorders: results in 14 patients. Sleep Med. 2003;4(4):281-284. doi:10.1016/s1389-9457(03)00072-8	2003	353	17.65	Humans
96	Sonnen JA, Larson EB, Crane PK, et al. Pathological correlates of dementia in a longitudinal, population-based sample of aging. Ann Neurol. 2007;62(4):406-413. doi:10.1002/ana.21208	2007	351	21.94	Humans
97	Mosconi L, Mistur R, Switalski R, et al. FDG-PET changes in brain glucose metabolism from normal cognition to pathologically verified Alzheimer’s disease. Eur J Nucl Med Mol Imaging. 2009;36(5):811-822. doi:10.1007/s00259-008-1039-z	2009	350	25	Humans
98	Irwin DJ, White MT, Toledo JB, et al. Neuropathologic substrates of Parkinson disease dementia. Ann Neurol. 2012;72(4):587-598. doi:10.1002/ana.23659	2012	350	31.82	Humans
99	Kosaka K. Diffuse Lewy body disease in Japan. J Neurol. 1990;237(3):197-204. doi:10.1007/BF00314594	1990	350	10.61	Humans
100	Nakashima-Yasuda H, Uryu K, Robinson J, et al. Co-morbidity of TDP-43 proteinopathy in Lewy body related diseases. Acta Neuropathol. 2007;114(3):221-229. doi:10.1007/s00401-007-0261-2	2007	350	21.88	Humans

### Year and country of publication

The 100 studies were conducted over a period of 39 years, from 1980 to 2019 (Fig. 1). The greatest number of studies were published in the year 2000. These articles originated from 17 countries (Fig. 1B); the USA was the largest contributor (*n* = 31), followed by the United Kingdom (*n* = 14).

### Authors and H index

A total of 759 authors contributed to these articles, with each article having a median of 8 authors (IQR 7, range 1–55). Authors with 4 or more articles in the top 100 most cited articles have been listed in Table 2. The greatest number of articles were co-authored by John Q Trojanowski (*n* = 9) and Ian G. Mckeith had the greatest number of articles as the first author (*n* = 5). For the top authors, there was a significant moderately positive correlation between the H indices and the number of their articles in the list (*P* = 0.008, *r* = 0.601).

**Table 2:** List of the top contributing authors on dementias with Lewy bodies

Author	Total articles	Authorship position			H index
		First	Last	Other	
Trojanowski JQ	9	0	3	6	226
Masliah E	8	2	3	3	172
McKeith I	8	5	0	3	107
Lee VM	8	0	3	5	199
Boeve BF	6	3	0	3	148
Perry H	5	1	0	5	98
Ballard C	4	2	0	2	116
Goedert M	4	0	3	1	133
Hurtig HI	4	1	0	3	58
Iranzo A	4	3	0	1	71
Jakes R	4	0	0	4	64
Lees A	4	0	1	3	161
Montine TJ	4	0	2	2	114
O’Brien J	4	0	3	1	116
Perry E	4	0	4	0	100
Rockenstein E	4	1	0	3	80
Silber MH	4	0	1	3	58
Fairbairn A	4	0	0	4	38

### Journals and institutions

The top 100 most-cited articles were published in 40 journals with IFs ranging from 1.1 to 168.9 (Table 3). Neurology (*n* = 12) made the biggest contribution, followed by Brain (*n* = 11). The greatest number of articles were published in journals related to neurology/neuroscience (*n* = 18), followed by medicine (*n* = 5), multidisciplinary (*n* = 5), biochemistry (*n* = 4), psychology/psychiatry (*n* = 3), pathology (*n* = 2), nuclear medicine (*n* = 2), and basic sciences (*n* = 1). A significant mild positive correlation was observed between the number of citations of an article with the IF of its journal (*P* = 0.016, *r* = 0.241) and the average citations per year of an article with the IF of its journal (*P* = 0.000, *r* = 0.419).

**Table 3:** Distribution of top 100 most-cited original articles by journals

Journal	Number of articles	Impact factor
*Neurology*	12	9.9
*Brain*	11	14.5
*Annals of Neurology*	6	11.2
*Journal of Biological Chemistry*	6	4.8
*Neuron*	5	16.2
*Acta Neuropathologica*	4	12.7
*Journal of Neuroscience*	4	5.3
*American Journal of Pathology*	3	6
*JAMA Neurology (Archives of Neurology)*	3	29
*Proceedings of the National Academy of Sciences of the United States of America*	3	11.1
*Science*	3	56.9
*The Lancet Neurology*	3	48
*FASEB Journal*	2	4.8
*Journal of Neurochemistry*	2	4.7
*Journal of Neurology Neurosurgery and Psychiatry*	2	11
*Journal of Neuropathology and Experimental Neurology*	2	3.2
*Lancet Neurology*	2	48
*Nature*	2	64.8
*Nature Communications*	2	16.6
*Sleep Medicine*	2	4.8
*Alzheimer Disease and Associated Disorders*	1	2.1
*Archives of General Psychiatry/JAMA Psychiatry*	1	25.8
*British Medical Journal*	1	105.7
*Clinical Neuropathology*	1	1.1
*European Journal of Nuclear Medicine and Molecular Imaging*	1	9.1
*FEBS Letters*	1	3.5
*International Journal of Geriatric Psychiatry*	1	4
*Journal of Comparative Neurology*	1	2.5
*Journal of Experimental Medicine*	1	15.3
*Journal of Neurology*	1	6
*Journal of Neuroscience Research*	1	4.2
*Journal of Nuclear Medicine*	1	9.3
*Journal of the Neurological Sciences*	1	4.4
*Lancet*	1	168.9
*Molecular Neurodegeneration*	1	15.1
*Nature Cell Biology*	1	21.3
*Nature Genetics*	1	30.8
*Nature Neuroscience*	1	25
*PLoS ONE*	1	3.7
*Psychological Medicine*	1	6.9
*The Journal of Pathology*	1	7.3

A wide range of institutions contributed to the top 100 cited articles, with institutions having more than 1 articles listed in Table 4. The greatest contribution was by the University of California (*n* = 7).

**Table 4:** Institutions contributing multiple articles to the top 100 most-cited original articles.

Institution	Number of articles
University of California	7
Harvard Medical School, Massachusetts General Hospital	6
Newcastle General Hospital	6
Mayo Clinic	4
University of Cambridge	4
University of Pennsylvania	4
Hospital Clínic de Barcelona	3
University of Newcastle upon Tyne	3
Johns Hopkins University School of Medicine	2
Medical Research Council, Laboratory of Molecular Biology, Cambridge, UK	2
New York University School of Medicine	2
Rush University Medical Center	2
Sun Health Research Institute	2
University of Tokyo	2

### Gender of authors

The first authors of the top 100 original articles were mostly males (*n* = 67), with a small proportion of females (*n* = 21). We were unable to retrieve the gender of the first authors in 12 articles. A significant relationship was present between the gender of the first authors and citations per year of each article (*P* = 0.021). The mean rank of citations per year was higher in articles where the first author’s gender was female compared to male (55.71 versus 40.99). The last authors of the top 100 original articles were also predominantly male (*n* = 70), with only a small representation of females (*n* = 19). We could not retrieve the gender of 11 senior authors.

### Conflict of interest and funding

Only 9 of the original studies disclosed a conflict of interest. Most of the original articles received funding (*n* = 61). No significant association was observed between the presence of a conflict of interest or funding with the total number of citations or the citations per year of the articles.

### Review studies

The top 15 review articles are mentioned in Table 5. These were published between the years 1988 and 2017. The most cited review article was “Diagnosis and management of dementia with Lewy bodies: third report of the DLB Consortium” published in Neurology in 2005. The review articles with the greatest number of citations per year was “MDS clinical diagnostic criteria for Parkinson’s disease” published in Movement Disorders in 2015.

**Table 5:** Top 15 review articles on dementias with Lewy bodies

Rank	AMA citation	Year	Cited by	Average citations per year
1	McKeith IG, Dickson DW, Lowe J, et al. Diagnosis and management of dementia with Lewy bodies: third report of the DLB Consortium [published correction appears in Neurology. 27 December 2005;65(12):1992]. Neurology. 2005;65(12):1863-1872. doi:10.1212/01.wnl.0000187889.17253.b1	2005	4287	238.17
2	Emre M, Aarsland D, Brown R, et al. Clinical diagnostic criteria for dementia associated with Parkinson’s disease. Mov Disord. 2007;22(12):1689-1837. doi:10.1002/mds.21507	2007	2227	139.19
3	Dani JA, Bertrand D. Nicotinic acetylcholine receptors and nicotinic cholinergic mechanisms of the central nervous system. Annu Rev Pharmacol Toxicol. 2007;47:699-729. doi:10.1146/annurev.pharmtox.47.120505.105214	2007	980	61.25
4	Heneka MT, Kummer MP, Latz E. Innate immune activation in neurodegenerative disease. Nat Rev Immunol. 2014;14(7):463-477. doi:10.1038/nri3705	2014	963	107.00
5	Postuma RB, Berg D, Stern M, et al. MDS clinical diagnostic criteria for Parkinson’s disease. Mov Disord. 2015;30(12):1591-1601. doi:10.1002/mds.26424	2015	3784	473.00
6	McKeith IG, Galasko D, Kosaka K, et al. Consensus guidelines for the clinical and pathologic diagnosis of dementia with Lewy bodies (DLB): report of the consortium on DLB international workshop. Neurology. 1996;47(5):1113-1124. doi:10.1212/wnl.47.5.1113	1996	3688	136.59
7	Olanow CW, Tatton WG. Etiology and pathogenesis of Parkinson’s disease. Annu Rev Neurosci. 1999;22:123-144. doi:10.1146/annurev.neuro.22.1.123	1999	1185	49.38
8	Goedert M. Alpha-synuclein and neurodegenerative diseases. Nat Rev Neurosci. 2001;2(7):492-501. doi:10.1038/35081564	2001	1142	51.91
9	O’Brien JT, Erkinjuntti T, Reisberg B, et al. Vascular cognitive impairment. Lancet Neurol. 2003;2(2):89-98. doi:10.1016/s1474-4422(03)00305-3	2003	1092	54.60
10	Ransohoff RM. How neuroinflammation contributes to neurodegeneration. Science. 2016;353(6301):777-783. doi:10.1126/science.aag2590	2016	1263	180.43
11	Jucker M, Walker LC. Self-propagation of pathogenic protein aggregates in neurodegenerative diseases. Nature. 2013;501(7465):45-51. doi:10.1038/nature12481	2013	1174	117.40
12	Gibb WR, Lees AJ. The relevance of the Lewy body to the pathogenesis of idiopathic Parkinson’s disease. J Neurol Neurosurg Psychiatry. 1988;51(6):745-752. doi:10.1136/jnnp.51.6.745	1988	2930	83.71
13	McKeith IG, Boeve BF, Dickson DW, et al. Diagnosis and management of dementia with Lewy bodies: Fourth consensus report of the DLB Consortium. Neurology. 2017;89(1):88-100. doi:10.1212/WNL.0000000000004058	2017	2355	392.50
14	Jenner P, Olanow CW. Oxidative stress and the pathogenesis of Parkinson’s disease. Neurology. 1996;47(6 Suppl 3):S161-S170. doi:10.1212/wnl.47.6_suppl_3.161s	1996	902	33.41
15	Lashuel HA, Overk CR, Oueslati A, Masliah E. The many faces of α-synuclein: from structure and toxicity to therapeutic target. Nat Rev Neurosci. 2013;14(1):38-48. doi:10.1038/nrn3406	2012	1148	104.36

## Discussion

Our bibliometric analysis of the 100 most-cited articles on LBD demonstrates a macroscopic view of contemporary trends and developments in this field. Lewy bodies were first discovered in 1912,[10] however, studies within the top 100 most-cited list were not conducted until 1980. Even then, it was not until the 1990s that protein α-synuclein, a key constituent of Lewy bodies, was described[11]. This may serve to explain the surge in research activity in the late 1990s. Bibliometric analyses on neuro-oncology have demonstrated a similar 1990s peak^[12,13]^. We found that the period with the most cited articles on LBD was from 1980 to 2019. The paucity of literature before the 1980s also indicates that much of the developments in neuroimaging and therapeutic regimens for LBD have emerged recently.

Between the years 2000–2009, research in this domain flourished with 52 publications during this period. While Alzheimer’s disease (AD) remains the most prevalent type of dementia, the financial burden posed by LBD is much higher[14]. The field has seen a surge of interest in recent years, with multiple systematic reviews and meta-analyses published since 2015.^[15-17]^ However, like most bibliometric analyses, we noted a downward trend in publications in recent years. Newer articles are often cited less during their earlier years given the shorter duration to accumulate citations[13]. For a more accurate evaluation of a publication’s scientific value and long-term impact, we organized articles by citations per year. Based on this, the most cited study was published comparatively recently in 2019 by Kim *et al*[18]. In this paper, the authors tested the Braak hypothesis of the retrograde spread of α-synucleinopathy pathology from the gastrointestinal tract to the brain via the vagus nerve in a novel gut-to-brain α-syn transmission mouse model and presented their results. A recent analysis by the Lewy Body Dementia Project Team has also revealed a growing interest in alpha-synuclein since 2016 among LBD researchers[19]. This highlights the evolving focus on key pathological mechanisms within the LBD research community. Our analysis further revealed that the majority of studies, over 70%, focused on human subjects. This indicates a significant advancement in LBD research, transitioning from animal studies to a more comprehensive approach involving human subjects and ex-vivo studies.

While LBD is primarily discussed in neurology, top-cited articles span various disciplines like medicine, biochemistry, psychology, pathology, and nuclear medicine. This challenges the Bradford law which states that to maximize the impact of a study within a particular field, authors tend to submit their articles to a few specialty-specific journals[20]. This implies that LBD-related research attracts a diverse readership beyond neuroscience. Moreover, we found a significant positive correlation between the number of citations of an article and its journal’s impact factor, which reinforces the concept that the prestige of a journal directly influences the impact its articles will have. Despite this finding, we find it pertinent to highlight that several of the papers in the top 100 list were published in relatively low impact factor journals. While the journal’s prestige is certainly important, it’s not the only factor, and most likely not even the main factor, behind a paper’s impact.

Our results showed that 18 individuals authored 4 or more articles in the top 100 list. Moreover, we found a significant moderately positive correlation between an author’s H index and the number of their articles in the list, indicating the dominance of a select few renowned individuals in the contemporary field of LBD. Comparatively, analyses conducted in cardiology subspecialties mirror our findings^[21,22]^. When stratified by gender, significant differences were uncovered with nearly two-thirds of the first and last authors in the top 100 list being males. This gender skew aligns with findings from previous studies indicating a correlation between the senior author’s gender and the likelihood of the first author also being of the same gender[23]. Furthermore, we found a significant association between the gender of the first authors and citations per year of each article, underscoring the broader implications of gender representation in scholarly impact. However, as a control group was not incorporated into the analysis, it is unclear whether the perceived gender differences are exclusive to this field or are simply a reflection of the broader differences seen in all fields of science.

When categorizing articles by country of origin, the United States emerged as the foremost contributor to LBD research, aligning with prior bibliometric studies. This dominance is likely due to substantial government funding for brain research, which is more than double that of Europe[24]. On the other hand, the representation of Asian and South American countries in the top 100 list was minimal^[25,26]^. In developing nations, while research related to communicable diseases saturates most of the literature, non-communicable diseases like LBD are starkly underrepresented. Furthermore, various institutions contributed to the top 100 cited articles. Of these, eight institutes were based in the United States and four in the United Kingdom. Additionally, over two-thirds of the original articles received funding. Despite the increasing interest of the scientific community in LBD, research in this area lacks substantial support – with fewer government grants allocated[19] and less than one-eighth of the funding per patient – leading to a disproportionately small number of publications[27]. Furthermore, LBD remains significantly behind in therapeutic development in the pharmaceutical and biotechnology industries.^[28-30]^ The primary funder of LBD research in the United States has been the NIH, with nearly half of NIH-funded projects focusing on the molecular mechanisms of the disease[19]. In addition, only a single randomized controlled trial (RCT) made it to the top 100 list, while there is a preponderance of wet lab studies. RCTs require much more resources and funding, but ultimately do not reach high citation counts. This might be one of the factors leading to most of the funding going towards molecular mechanisms.

Our findings should be interpreted with some limitations. Firstly, our analysis relied solely on one database, Scopus, potentially excluding articles not indexed by it, potentially introducing selection bias. Additionally, citation counts and H-indexes may differ from those in other databases, impacting the analysis. Secondly, Scopus may overlook articles published before the advent of computerized systems in the 1980s[8]. Thirdly, concerns about self-citations potentially biasing results have been noted, particularly when a few authors dominate high-frequency citations. However, research has indicated that self-citations have minimal impact on bibliometric analyses[31]. Fourthly, citation counts are not always representative of a paper’s impact and significance. This is true in our analysis as well, evidenced by the fact that the article, “McKeith IG, Perry RH, Fairbairn AF, Jabeen S, Perry EK. Operational criteria for senile dementia of Lewy body type (SDLT). Psychol Med. 1992;22(4):911-922. doi:10.1017/s0033291700038484”, was arguably the first to describe LBD, but ranked only 72 on the list. Fifthly, we used pronouns as a surrogate for gender, the two may not necessarily align in all cases and some people’s genders remained unidentified due to this approach. Sixthly, our eligibility criteria dictated the inclusion of all papers which included patients with LBD; this inadvertently led to the inclusion of a few all-cause dementia papers only marginally related to LBDs, which might not provide any specific insights. Lastly, very recent articles with the potential for landmark status may not have been included due to the time required for citations to accumulate.

## Conclusions

While LBD has received some financial support over time, more comprehensive efforts are needed for the clinical evaluation and management. Research on LBD is predominantly centered in a few countries, highlighting the need for studies in other populations. There was a scarcity of RCTs among the highly cited articles, which also raises concerns for funding going towards molecular and lab studies. Additionally, our study identifies key research trends that can guide future investigations. Given the rising interest of researchers in LBD, it’s imperative to foster increased collaboration within the scientific community to address this pressing issue.

## Data Availability

The datasets generated during the current study are available upon reasonable request.

## Presentation

The abstract of this paper was presented at the 2024 UCL Neuroscience Symposium.
